# Prevention of Adult Colitis by Oral Ferric Iron in Juvenile Mice Is Associated with the Inhibition of the Tbet Promoter Hypomethylation and Gene Overexpression

**DOI:** 10.3390/nu11081758

**Published:** 2019-07-31

**Authors:** Chourouk Ettreiki, Abalo Chango, Nicolas Barbezier, Moise Coeffier, Pauline M Anton, Carine Delayre-Orthez

**Affiliations:** 1 PETALES Team, EA 7519 Transformations & Agro-Resources Research Unit, UniLasalle, F-60026 Beauvais, France; 2 INSERM UMR 1073, Normandie University, UNIROUEN, F-76183 Rouen, France

**Keywords:** ferric iron, intestinal inflammation, Th profile, Tbet, promoter methylation

## Abstract

Iron is an essential nutrient needed for physiological functions, particularly during the developmental period of the early childhood of at-risk populations. The purpose of this study was to investigate, in an experimental colitis, the consequences of daily oral iron ingestion in the early period on the inflammatory response, the spleen T helper (Th) profiles and the associated molecular mechanisms. Juvenile mice orally received microencapsulated ferric iron or water for 6 weeks. On adult mice, we induced a sham or experimental trinitrobenzene sulfonic acid (TNBS) moderate colitis during the last week of the experiment before sacrificing the animals 7 days later. The severity of the gut inflammation was assessed by macroscopic damage scores (MDS) and the myeloperoxidase activity (MPO). Th profiles were evaluated by the examination of the splenic gene expression of key transcription factors of the Th differentiation (*Tbet*, *Gata3*, *Foxp3* and *RORγ*) and the methylation of their respective promoter. While TNBS-induced colitis was associated with a change of the Th profile (notably an increase in the *Tbet/Gata3* ratio in the spleen), the colitis-inhibition induced by ferric iron was associated with a limitation of the splenic Th profiles perturbation. The inhibition of the splenic *Tbet* gene overexpression was associated with an inhibition of promoter hypomethylation. In summary, mice treated by long-term oral ferric iron in the early period of life exhibited an inhibition of colitis associated with the inhibition of the splenic *Tbet* promoter hypomethylation and gene overexpression.

## 1. Introduction

Early iron fortification of food is generalised in Western Countries to prevent any risk of anaemia. In general, the forms of supplementation are ferrous (Fe^2+^) but they are associated with frequent gastrointestinal side effects leading to poor compliance. In a previous study, we evidenced that the early administration of a new form of iron fortifier, lecithin bead microencapsulated ferric pyrophosphate (Fe^3+^), is more efficient in preventing colitis in adult mice than its ferrous counterpart [1]. One of the explanations of such an effect is that crucial events during the perinatal stage drive the modelling of the memory mechanisms of immunity response maturation to reach full functionality. The immune system is mostly defined by an organised collection of cells interestingly concurring with their physiological function and development [2]. The high specificity of these immune cells in defending responses and the vast variability of their phenotypes were for a long time explained by a remarkable programming process that happens during the early life stages [3,4,5]. Among the numerous cells belonging to the immune system, lymphocytes participate in the maturation of the organism and its adaptation, from the maternal environment to the external environment rich in multiple pathogenic substances. Indeed, during the prenatal period, the foetal immune system profile remains naïve except for a small number of T helper (Th) lymphocytes that are polarised into a T helper type 2 (Th2) profile, a situation required to protect the foetus from rejection [4]. After birth, depending on the types of exposures to environmental stimulations that promote progressive immune system expansion and the differentiation of the other profiles, the future immune profile is determined [3,4]. Under these conditions, specific factors operate to activate naive Th cells and determine the polarisation of the Th subsets. Specific antigen recognition and local circulating cytokine factors initiate the process of differentiation and progressive development of the other sub-populations of Th cells. The maintenance and the amplification of the expression of specific Th cytokine genes that qualify each Th pattern are preserved and regulated by transcription factors. The key transcription factors for the cytokine signatures of Th1, Th2, Th17 and Treg subpopulations are, respectively, *Tbet*, *Gata3*, *RORɣ* and *Foxp3* [6]. Positive or negative interactions between these transcription factors have been shown, thus, giving the lead to one of the Th profiles or modulating the inflammatory response. For example, it was shown that T-bet is a key modulator of IL-23-driven colitogenic responses in the intestine [7]. Various transversal signalling pathways are also in direct connection with the promoter and the control locus regions of these transcription factors inducing the repression or the activation of gene expression as well as epigenetic modifications. Furthermore, studies have demonstrated epigenetic control of the expression of cytokines and key transcription factor genes involved in Th cells development [8,9,10]. In fact, these epigenetic regulations are also described as a bridge between the genotype and phenotype through changes influenced by the environment [11,12]. This refers to an adaptive response of cells towards various changes and events enounced during life (stress, specialisation and differentiation, dependency, etc.) [11,13], which happen through various processes such as DNA CpG islands methylation, histone modification, and small regulatory RNAs, as cellular responses to signals from the environment [14]. A relevant example that induced epigenetic changes in response to environmental signals is the differentiation of multipotent naive Th lymphocytes into distinct subpopulations [8,15]. In fact, during the perinatal period, epigenetic processes mediate massively temporary or permanent specific gene activation or repression depending on the environmental context and, therefore, preset the future Th profile [10,15]. Consequently, the exposition to abnormal environmental conditions may induce some modifications of the epigenetic profile and leads to a Th imbalance and a pathogenic immune profile observed during several inflammatory immune-related diseases [16,17].

Among the environmental factors, diet exposure considerably contributes to the modulation of the orientation of the immune profile and, more particularly, during the early postnatal period, which is a critical time window for epigenetic dysregulation. The literature has described the ability of nutrition, in the very early postnatal period, to induce epigenetic regulations leading to the beneficial or deleterious profile [18,19]. Some of these molecular mechanisms have been described. For instance, it has previously been determined that in mammalians, the one-carbon metabolic pathway depends mainly on the influence of the nutrient substrate (methyl donors) on the CpG islands methylation [20,21]. Other studies have pointed out a direct association between some micronutrients and CpG islands methylation linked to chronic diseases development [22,23]. However, the incidence of iron ingestion on the gene methylation processes has not yet been investigated.

In a previous study, we demonstrated that the early ingestion of microencapsulated ferric iron prevents microbiota dysbiosis and colitis in adult mice [1]. To further understand this effect, the current study aimed to provide evidence for the role of the immune system and, notably, its splenic Th profile modulation after iron ingestion during the perinatal period. Thus, the current study aimed to provide evidence whether the inhibition of colitis by early ingestion of this ferric iron is associated with modulation of (1) the spleen expression of transcription factors related to the Th profile and (2) the methylation on CpG islands present into promotor regions of these transcription factors. Investigations were carried out on mice supplemented from the juvenile period to adulthood with the microencapsulated ferric iron formulation and submitted to the Th1 model of inflammatory digestive pathologies at adulthood. This mice model is widely recognised for reproducing the immune and inflammatory reactions observed in humans. The impact of iron ingestion on the inflammatory reaction and on the genetic and epigenetic regulations of transcription factors involved in the immune orientation was investigated at adulthood.

## 2. Materials and Methods

### 2.1. Chemicals

Lipofer^®^, lecithin bead microencapsulated ferric pyrophosphate, is a gift from Lipofoods Ltd., Barcelona, Spain. TNBS, human myeloperoxidase, and bovine serum albumin (BSA) were obtained from Sigma-Aldrich SA, St Quentin Fallavier, France.

### 2.2. Animals

Four-week-old male BALB/c mice (n = 54) were obtained from HARLAN Laboratories, Gannat (France). All animals were housed in stainless steel cages (4–5 mice/cage) under a controlled temperature (21 ± 1 °C) and 12 h light-dark cycles in compliance with the undergoing legislation and recommendations. They had free access to food (A04, SAFE, Epinay sur Orge, France) and water throughout the study. Experimental protocols were approved by the Ethics Committee (No. CEEA116). Animal care, handling and experimentation complied with the EU guide for use of laboratory animals and the undergoing French legislation.

### 2.3. Experimental Procedure

**Figure 1:**
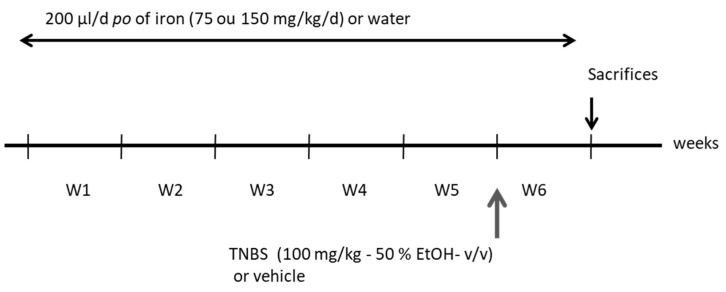
The experimental design of the 6-week-long study (W1 to W6). Animals received 200 µL/day of either ferric iron (75 or 150 mg/kg/day *po*) or water during the 6 weeks. Animals received trinitrobenzene sulfonic acid (TNBS) (100 mg/kg) or its vehicle during week 6 and were kept for seven days before sacrifice.

Three groups of 18 mice were used. They received ferric iron (75 or 150 mg/kg/day *po* - Lipofer^®^) or water daily during the 6 weeks. Each group was split into 2 sub-groups (n = 9 mice each). For each iron treatment, the first group was submitted to the TNBS-induced colitis during the last week of the experiment and the second group only received the vehicle (water in 50% ethanol). Weight variations of all animals were recorded during the procedure (Figure 1). At the end of the experiment, the mice were anaesthetised with sodic pentobarbital (60 mg/kg). Blood was drawn from the abdominal vein for seric immunoassays. Sera were collected by centrifugation for 15 min/7000 g/4 °C and stored at –20 °C until processed. Then, the mice were sacrificed and the colon and spleen tissues were harvested, snap-frozen in liquid nitrogen and stored at –80 °C until further determination.

### 2.4. Experimental Inflammation

Animals were fasted overnight prior to colitis induction to offer better contact with the colonic lumen, but were allowed free access to water. Briefly, mice were anaesthetised with a mixture of ketamine/xylazine (12.5 mg/kg) and instilled with trinitrobenzene sulfonic acid (TNBS) diluted in 50% of ethanol (*v*/*v*) (100 mg/kg–25 µL), as previously described [1]. The control mice were instilled with the vehicle, i.e., water and ethanol (50% *v*/*v*).

### 2.5. Assessment of Colitis

#### 2.5.1. Macroscopic Lesions

After sacrifice, the colon was removed immediately and the severity of the colonic mucosal alteration was determined according to a modified scale by Wallace et al., [24]. Briefly, the determination of the inflammatory damages was based on the presence of mucosal hyperaemia and bowel wall thickening, the presence and extent of ulceration and necrosis, and the event of adhesions and diarrhoea. Scores were established from normal appearance (0) to severe damage (10).

#### 2.5.2. Myeloperoxidase (MPO) Assay

MPO activity, a marker of neutrophils tissular infiltration, was measured in the pieces of the colon adjacent to the instillation point as previously described [1] and according to Bradley et al., [25]. Briefly, sample homogenisation was followed by protein isolation and the measurement of MPO activity. Human MPO from purified neutrophils was used as a standard. The absorbance was measured after 10 min of incubation with H2O2 and chloride at 450 nm. The total protein content was assessed from the supernatants based on Lowry’s method (Bio Rad DC Protein Assay, Marnes-la-Coquette, France).

### 2.6. Splenic Th Transcription Factors Gene Expression

RNA extraction: The total RNAs were extracted using the RNeasy Mini plus Kit (Qiagen, Courtaboeuf, France). The spleen was homogenised in the manufacturer’s RLT buffer using a Tissue Lyser apparatus (Qiagen, Courtaboeuf, France). Then, the RNA was automatically extracted from the homogenate using a Qiacube machine (Qiagen, Courtaboeuf, France). Concentrations were determined based on the NanoDrop technology (Hellma TrayCell) using a Biophotometer apparatus (Eppendorf, Montesson, France). Absorbance ratios at 260/280 nm and at 260/230 nm were used to quantify and assess the purity of the DNA samples.

Reverse transcription: RNA samples were converted to cDNA using the QuantiTect Reverse Transcription Kit (Qiagen, Courtaboeuf, France). Briefly, samples were incubated with a genomic DNA wipe-out buffer at 42 °C for the effective elimination of any genomic DNA. RNA samples were submitted to reverse transcription using a master mix prepared with the Quantiscript RT buffer and the RT Primer Mix (QuantiTect Reverse Transcription Kit, Qiagen, Courtaboeuf, France). Reverse transcription was activated 20 min at 42 °C and then inactivated 3 min at 95 °C.

Quantitative PCR (qPCR): Primers for *Tbet*, *Gata3*, *Foxp3*, *RORγ* and for the housekeeping gene *Gapdh* were designed using the Primer Express Software (PE-V2.0-Applied Biosystems, Illkirch, France) based on the RNA messenger transcripts published in the database of the NCBI GenBank (Table 1).

qPCR was performed on the ABI Prism 7300 sequence detector system (Applied Biosystems) using a Sybr Green PCR master mix. A total of 5 µL of each cDNA (20 ng/µL) was amplified in 20 µL of PCR mixture containing a 2X SYBR Green master mix and 300 nM of each primer. After the activation cycle at 95° for 3 min, forty amplification cycles at 95° for 3 sec and 61° for 30 sec were established. Non-specific amplification was confirmed by the analysis of the melting curve. qPCR was determined based on the threshold cycle number (Ct). The relative quantification of gene expression was calculated by the comparative Ct method (2^−∆∆Ct^) and was normalized by the *Gapdh* reference gene according to the method by Livak and collaborators [26], where ∆∆Ct = (Ct of target gene − Ct of the reference gene) of the treated condition was normalized by the ∆∆Ct of the control condition.

### 2.7. Methylation Status of CpG Islands

DNA extractions: DNA isolations from the spleens were performed using the QIAGEN Blood & Tissue Kit (Qiagen, France). Briefly, samples weighing less than 10 mg were placed in 2 mL microcentrifuge tubes with 180 µL of the manufacturer’s ATL buffer and then homogenized in the Tissue Lyser. Twenty microlitres of proteinase k were added before incubation (3 h, 56 ℃). Then all of the homogenates were automatically extracted using a Qiacube.

**Figure 2:**
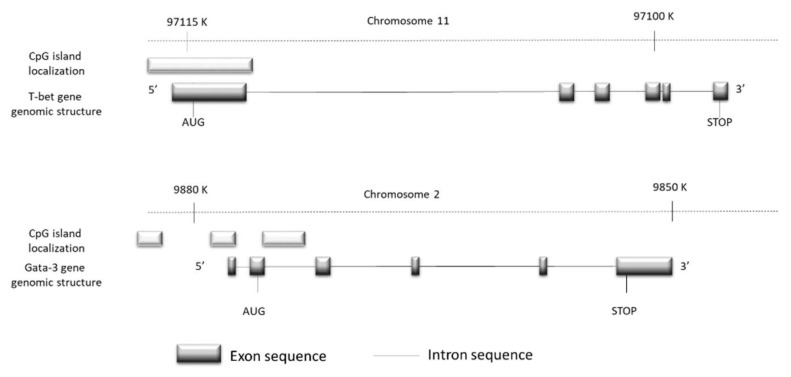
The representation of the promotor genomic regions of *Tbet* and *Gata3* showing the localization of all CpG regions present into promotor regions of these transcription factors.

Methylation profile analysis: the evaluation of the methylation status of specific CpG islands of the *Tbet* and *Gata3* promoters (Figure 2) were realised with the help of the Methyl-Profiler™ DNA Methylation Enzyme Kit (Sabioscience, France) that contains all necessary components for the cleavage of methylated and unmethylated DNA according to the manufacturer’s recommendations. This is a classical method based on the principle of amplification of different DNA regions corresponding to CpG island regions involved in the regulation of the initiation of genes transcription. These regions have been submitted before amplification to digestion by specific restriction enzymes that are or are not sensitive to the presence of methyl groups. Briefly, DNA was digested in four equal reactions: (1) Mock Digest (Mo) in which no enzyme was added to the reaction buffer. This condition is a negative background. (2) Methylation Sensitive Digest (Ms), revealing a single cleavage with a methylation-sensitive enzyme in order to digest unmethylated and partially methylated DNA. (3) Methylation Dependent Digest (Md), revealing a single cleavage with a methylation-dependent enzyme aiming at preferentially digesting methylated DNA. The remaining unmethylated DNA was detected by qPCR. (4) Double Digest (Msd), in which the two enzymes were added in the double digest, and in which all DNA molecules (both methylated and unmethylated) were digested. This last condition corresponds to a positive background. Real-time PCR was performed according to the abovementioned conditions and a total volume of reaction of 27.5 µL was used. For 5 µL of each digest product, we added 1.1 µL of specific primers delivered with the kit, 13.5 µL of SYBR^®^ Green qPCR Master Mix (Qiagen, France), and adjusted the volume with RNase free water.

The comparison between the presence or absence of different amplification products allowed us to identify the region with the methyl groups. Indeed, in nonsensitive conditions, these regions were cut by the restriction enzyme nonsensitive to the methylation and were not amplified by qPCR, whereas they were not cut by the sensitive restriction enzyme and consequently were not amplified by qPCR.

### 2.8. Statistical Analysis

The results are presented as Mean ± SEM or Tukey box-plot. Statistical analysis was performed using the Graph Pad PRISM Software (V5.0). Macroscopic damage and anaphylactic response scores were compared using the Mann–Whitney test for non-parametric data. For all of the other parameters studied, the data were submitted to an ANOVA test followed by the post-test of Tukey for unpaired data. A value of *p* < 0.05 was considered to be significant.

## 3. Results

### 3.1. Phenotypic Parameters of Colitis after Microencapsulated Ferric Iron Supplementation

Colitic mice groups presented the general symptoms visualised during colitis [27]. Temporal evaluation of the corporal weight evolution showed significant weight loss consecutively to TNBS instillation (Table 2) associated with diarrhoea and moderate hyperaemic ulceration which are classical criteria used for the macroscopic assessment of lesions. MPO activity from colonic tissue was significantly increased (*p* < 0.001) compared to the controls, suggesting the increase of the inflammatory status (Table 2). Mice submitted to colitis and orally treated with ferric iron (Iron75-TNBS and Iron150-TNBS) exhibited a significant limitation of their pathologic symptoms at both the two doses of iron administered. This effect was more marked with a dose of 150 mg/kg/day. Weight gains were maintained even during the inflammatory periods. MPO activity and macroscopic damages were nearly normalised (Table 2).

### 3.2. Beneficial Effect of Iron in the Pathologic Th1 Immune Orientation:

**Figure 3:**
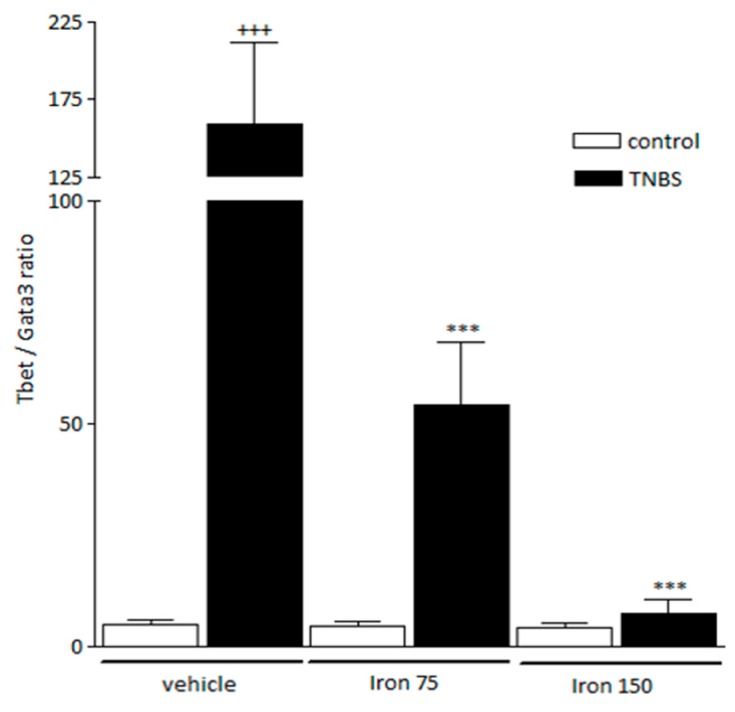
The impact of iron supplementation on the *Gata3* and *Tbet* gene expression ratio in the spleen. Data are expressed as Mean ± SEM. *** Significantly different (*p* < 0.001) from vehicle-treated TNBS mice. +++ Significantly different (*p* < 0.001) from vehicle-treated control mice. White column: Control mice, Black column: TNBS-treated mice. TNBS instillation resulted in an increase of the *Tbet/Gata3* ratio. By contrast, ferric iron ingestion dose-dependently limited the *Tbet/Gata3* ratio increase in TNBS-treated mice.

**Figure 4:**
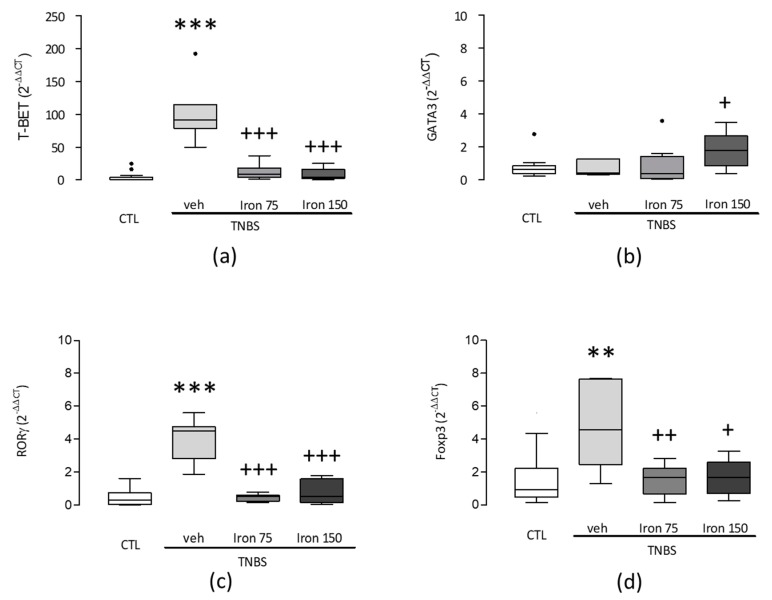
The impact of iron supplementation on the *Tbet* (**a**), *Gata3* (**b**), *RORɣ* (**c**) and *Foxp3* (**d**) gene expressions in the spleen. Data are expressed as a Tukey boxplot with a median. Each small black dot (•) represents individual data that is not included between the whiskers. **, *** significantly different (*p* < 0.01 and *p* < 0.001, respectively) from the controls (CTL). +, ++, +++ significantly different (*p* < 0.05, *p* < 0.01 and *p* < 0.001, respectively) from the vehicle-treated TNBS mice (veh-TNBS). Groups: Controls (CTL), Vehicle-treated TNBS mice (veh-TNBS), Ferric iron (75 mg/kg/day *po*), TNBS-treated mice (Iron 75-TNBS), Ferric iron (150 mg/kg/day *po*) TNBS-treated mice (Iron 150-TNBS). *Tbet*, *RORɣ* and *Foxp3* are significantly increased in TNBS treated mice (**a**,**c**,**d**). In contrast, splenic gene expression of *Tbet* (**a**), *RORɣ* (**c**) and *Foxp3* (**d**) were significantly lower in mice supplemented by ferric iron. Splenic *Gata3* expression was also enhanced in mice treated with ferric iron (150 mg/kg/day *po*).

Examination of the *Tbet* and *Gata3* gene expressions in the spleen, directly related to naive T-cells development toward the Th pathway, confirmed the selective orientation according to the immune profile. Mice that were submitted to TNBS (Veh-TNBS) showed a higher value of *Tbet/Gata3* ratio in the spleen (158.9 ± 52.8 vs. 4.8 ± 1.2 in controls) (Figure 3). This enhanced ratio was due to a strong increase of *Tbet* expression and a non-significant slight decrease of *Gata3* in the spleen (103.2 ± 16.9 vs. 3.6 ± 1.6 and 0.4 ± 0.2 vs. 0.7 ± 0.2, respectively, by comparison to controls) (Figure 4a,b). By contrast, this ratio was dose-dependently lowered in animals submitted to iron supplementation during the juvenile period and to the experimental colitis at adulthood (54.1 ± 14.3 and 7.3 ± 3.3 in 75 and 150 mg/kg/day *po* colitic mice respectively) (Figure 3). At the two doses of iron used, this lowered ratio comes mainly from a strong reduced *Tbet* gene expression in the spleen (Figure 4a). At 150 mg/kg/day, this was reinforced by the increased *Gata3* gene expression in the spleen (Figure 4b). In non-colitic mice, ferric iron, whichever dose tested, did not change the levels of expression of *Tbet* or *Gata3* as compared to vehicle-treated mice (Figure 3).

Examination of RORγ and *Foxp3* gene expressions in the spleen was undertaken to evaluate the modulation of the immune orientation by the Th17 and Treg immune cells. Induction of TNBS colitis resulted in a significantly (*p* < 0.001 and *p* < 0.01 respectively) increased splenic gene expression of the both transcription factors, *RORγ* and *Foxp3* (4 ± 0.5 vs 1.1 ± 0.4 and 4.8 ± 0.1 vs 1.7 ± 0.4, respectively, by comparison to controls) (Figure 4c,d). Furthermore, these results were closely associated with the elevated *Tbet* profile observed. When compared to the veh-TNBS group, both groups of mice treated by the ferric iron supplementations (Iron75-TNBS and Iron150-TNBS) presented a significantly lower splenic gene expression of *RORγ* (0.5 ± 0.1 and 0.8 ± 0.2% in 75 and 150 mg/kg/day *po* colitic mice, respectively) and *Foxp3* (1.7 ± 0.4 and 2 ± 0.5 in 75 and 150 mg/kg/day *po* colitic mice, respectively) (Figure 4c,d).

### 3.3. Evaluation of Locus Specific Methylation in Tbet and Gata3 Gene Promoters

**Figure 5:**
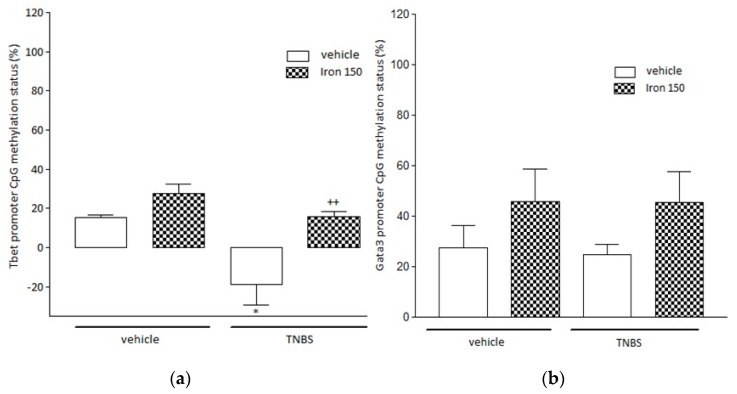
The impact of ferric iron supplementation on the *Tbet* (**a**) and *Gata3* (**b**) promoter CpG methylation status (%). Data are expressed as Mean ± SEM. * Significantly different (*p* < 0.05) from vehicle-treated mice. ++ Significantly different (*p* < 0.01) from vehicle-treated TNBS mice. White columns: vehicle-treated mice; squared columns: ferric iron (150 mg/kg/g *po*) treated mice. TNBS instillation resulted in a significant reduction (*p* < 0.001) of the *Tbet* promoter CpG methylation status (A) which was reversed by iron treatment (**a**). By contrast, we did not observe any modification of the methylation status of the promoter region of *Gata3* (**b**) regardless of whichever treatment was considered.

In colitic mice (Veh-TNBS), we evidenced a significant (*p* < 0.001) hypomethylation of the *Tbet* gene promoter region (–18.7 ± 10.5 vs. 15.4 ± 1.2% in controls) (Figure 5a) associated with the non-significant hypermethylation of the *Gata3* gene promoter region (Figure 5b) in the spleen. By contrast, we observed a significant (*p* < 0.01) limitation of the hypomethylation of the *Tbet* gene promoter region in Iron150-TNBS mice (15.7 ± 2.7%) (Figure 5a). In non-colitic animals, ferric microencapsulated iron did not change the methylation profile of either the *Tbet* (Figure 5a) or *Gata3* (Figure 5b) promoter regions.

## 4. Discussion

In addition to our first works on iron oral supplementation [1], this present study clearly illustrates the incidence of daily oral iron ingestion during the early period of life on the splenic Th profile and colitis symptoms at adulthood. Others studies have described that the immune profile of adults is in direct correlation with its early programming [3,4,5]. Our results are in accordance with the view of an intimate relationship between T-helper development and epigenetic regulation [8,9,10,28]. The study was aimed at identifying the effect of oral ferric iron supplementation in a model of Th1-colitis on the immune profile by studying the splenic expression of Th transcription factors and the accessibility of their promoters.

In order to address this point, we first confirmed the pathological status of the animal model. Mice submitted to the TNBS developed colitis, as evidenced by the analysis of the macroscopic lesions and MPO activity that are classical inflammatory parameters assessed to confirm inflammatory reactions. In fact, similar reports have been previously described in the literature and are in agreement with an increase of the inflammatory response associated with the TNBS-induced colitis model [29,30]. Significant weight variations and the macroscopic damage score of Wallace are two relevant parameters that easily reveal the presence of the intestinal physiological perturbations [23]. MPO activity, which reflects the level of neutrophil infiltration, is also usually used for determining colitic severity. In the presence of ferric iron, we confirmed the protective and dose-dependent effect of both doses of ferric pyrophosphate (75 and 150 mg/kg/day *po*) on the moderate colitis. 

We also analysed the incidence of iron supplementation on the modulation of the Th profile induced by a moderate TNBS colitis. Indeed, the literature has described that this experimental colitis response was being driven by a Th1 immune response (for review, see Reference [31]). In our experiment, seven days after inducing a moderate inflammatory response, the analysis of *Tbet* and *Gata3* expression in the spleen confirmed the Th1 profile, but also the main role of *Tbet* in the colitis, as illustrated by the strong increase of the *Tbet* expression (Figure 4a). The high *Tbet/Gata3* ratio also reflected the dominance of the Th1 lymphocytes polarisation in the colitic mice as already described [32,33]. Additionally, although the *Tbet* and *Gata3* transcription factors remain the two major factors evaluated for the Th profile, the other subpopulations of the Th cells also play a non-negligible role in the determination of the immune profile [34,35,36]. Thus, a massive Th17 immune response stimulation had previously been described mostly during the inflammatory responses, particularly reinforcing the Th1 immune response [37,38]. It was shown that colitis increases the expression of *RORγ* and *Foxp3* transcriptions factors [37,39]. Our results obtained with the TNBS group thus conform to the previous literature. However, these results were obtained from the whole splenic tissue. Splenic gene expressions of key transcription factors of Th differentiation (*Tbet*, *Gata3*, *Foxp3* and *RORγ*) are a reflection of the Th profile, but Th cell differentiation is regulated by the complex transcriptional network [40]. Consequently, further experiments will be needed to confirm these results by the flow cytometry analysis of Th subsets and single-cell RNA sequencing.

We evaluated the consequences of a repeated low dose supplementation in the juvenile period of a ferric iron form used as a food additive. Under these conditions, we evidenced a remarkable effect of this formulation (ferric micro-encapsulated iron) on the modulation of intestinal inflammation associated with the re-equilibration of the splenic gene expression of key transcription factors of Th differentiation. Our results corroborate other studies evidencing the physiological role of iron in relation to some inflammatory processes [41,42]. A preliminary study (same experimental setup) aiming at assessing the iron vehicle did not show any difference between water and lecithin bead microcapsules without ferric pyrophosphate. Both groups presented the same weight loss, i.e., –24% and –23% from the original weight in TNBS-treated mice receiving either water or lecithin bead microcapsules without ferric pyrophosphate, respectively, versus no weight loss in the control mice (Control: 23.6 ± 0.5 at D0-W6 vs. 23.9 ± 0.6 at D7-W6; TNBS+water: 22.1 ± 0.4 at D0-W6 vs. 16.9 ± 0.6 at D7-W6; TNBS+vehicle: 23.1 ± 0.3 at D0-W6 vs. 17.8 ± 0.4 at D7-W6). Moreover, animals presented similar significant macroscopic lesions since we scored them at 5.8 ± 0.7 and 6 ± 0.1 in TNBS-treated mice receiving either water or lecithin bead microcapsules without ferric pyrophosphate, respectively, versus 0 in the control mice (Figure S1). These results were not surprising since lecithin only counts for 0.12% of the total dry matter of the vehicle of Lipofer. Consequently, we evidenced that lecithin bead microcapsules alone do not have any effect on TNBS-induced colitis and, in turn, attributed all the effects to the iron form. The analysis of the transcription factors profile also showed the maintenance of the Th balance, notably due to a significant limitation of the substantial splenic expression of the *Tbet* gene and a non-significant slight activation of the splenic expression of the *Gata3* gene. Mice treated by early ferric iron supplementation, before inducing the colitis at adulthood, presented a normalisation of the Th17 and the Treg profiles. One hypothesis is that the Treg profile could be dependent on iron supplementation and that it may be explained, according to the study by Zeng et al., by the relationship between iron absorption and the phenomenon of anergy [43]. One other important result is the absence of a per se effect of early ferric iron supplementation in the animals, which plays in favour of the use of this microencapsulated form of ferric iron. Our results contrast with previous studies evidencing a negative effect [44,45,46]. The microencapsulation of iron may explain such a difference. Indeed, its kinetics of release and availability will necessarily be different from the usual iron supplementation.

Our results show that colitic mice treated with iron exhibited the modulation of splenic Th transcription factors gene expression. Since the modulation of the *Tbet* and *Gata3* expressions could be due to epigenetics, we chose to evaluate the incidence of daily oral iron ingestion on the methylation events of the promoter regions of *Tbet* and *Gata3*. In fact, it has already been established that some dietary components contribute to the epigenetic process modulating the immune system development [12] and condition and, at least in part, the establishment of intestinal homeostasis. From our results, chronic iron ingestion did not significantly affect the methylation level of the *GATA3* promoter CpG island studied. This could be in concordance with the slight modification of *GATA3* expression. However, as there are 3 putative CpG islands in the gene promoter, it will be interesting in the future to test methylation status of the remaining islands of this promoter. Another possibility would be to study the chromatin conformational modulation induced by histone (H3, H4) modifications as methylation or acetylation. Regarding the *Tbet* gene, after the induction of colitis, its promoter presented a decreased methylation (–18.7%) in the TNBS groups, supporting the hypothesis of an enhanced accessibility of the promoter for transcription factors and confirming the dominant expression of *Tbet* in this Th1 model observed above. Then, in mice supplemented with ferric iron, the epigenetic profile of the *Tbet* promoter was re-established since it presented a percentage of methylation comparable to one of the control groups (15.7% vs. 15.4%). One hypothesis, in adequation with others works that described the potential effect of particular nutrients modulating the epigenetic profile, could be that the iron may prevent the expansion of the *Tbet* gene expression observed in colitic mice [20]. Indeed, iron ingestion implicates a variation in the pool of free iron in the organism. Iron has the capacity to affect the intracellular redox state leading to a change in the activity of many enzymes including epigenetic enzymes. Additionally, as iron is a cofactor of many epigenetic enzymes, the variation of its concentration is able to modulate their activities [47]. Other hypotheses regarding the effects of chronic iron ingestion may be issued. Indeed, it was reported that iron is involved in the growth and function of immune cells and could modulate the Th1/Th2 ratio by a ROS-mediated mechanism [48,49,50], but contradictory results were found in different studies, possibly due to the form of iron [50,51].

The impact of the iron supplementation could also be indirect. In our previous study, we demonstrated that early ingestion of microencapsulated ferric iron prevents microbiota dysbiosis in adult mice [1]. Genetics, gut microbiota and the immune system are involved in the pathogenesis of IBD [52]. Intestinal microbiota plays a central role in the inflammation/tolerance balance by its influence on the Th profile, and gut microbiota dysbiosis could be a potential contributor to the inflammatory process [53,54]. Furthermore, it was shown that probiotics such as B. *infantis* could limit TNBS-induced colitis by inhibiting the Th1 and Th17 responses in mesenteric lymph nodes [55]. In our study, the inhibition of the Th1 and Th17 responses observed in the spleen could be the reflection of the modulation of the local intestinal Th response. We may hypothesise that the inflammation inhibition induced by iron is based on a double interconnected mechanism (gene expression and microbiota). Furthermore, it is known that innate immunity is also involved in the induction of inflammation. Here again, the impact of iron could be in two different ways, with the direct effect of iron on innate cells or the indirect effect by the modulation of microbiota. Finally, it was reported that iron could modulate gut barrier (epithelium and/or beneficial barrier commensal gut microbiota) and, thus, have an impact on colic inflammation, but this impact seems to be variable [56]. Confirmation of these hypotheses will require further investigations.

In conclusion, juvenile mice treated by daily oral ingestion of ferric iron presented a weaker alteration of the splenic gene expression of key transcription factors of Th differentiations in a Th1 colitis model associated with the inhibition of the *Tbet* promoter hypomethylation and the inhibition of adult colitis symptoms. If confirmed in other experimental models of inflammation, this supplementation could be of particular interest on the population prone to develop chronic inflammatory and autoimmune diseases during adulthood. 

## Figures and Tables

**Table 1:** The characteristics of the primer used for qPCR. *GAPDH* was used as housekeeping gene sequences are expressed from the 5′ to the 3′ region and were designed using primer express 3.

Gene Name	5′ → 3′ Sequence	Accession Number
*Tbet*	For-TGC CTA CCA GAA CGC AGA GA	NM_019507
	Rev-CGG AAT CCT TTG GCA AAG G	
*Gata3*	For-GAC CCC TTC TAC TTG CGT TTT TC	NM_008091
	Rev-ACA TTT TGC TTT CTG CCT TCA AA	
*RORɣ*	For-GCT CTG CCC CCA GTG ACA	NM_011281
	Rev-TGC AAC CTC AAG GAA GAG ATT G	
*Foxp3*	For-CCT CTA GCA GTC CAC TTC ACC AA	NM_001199347
	Rev-TCA ATA CCT CTC TGC CAC TTT CG	
*Gapdh*	For-CTG CCA AGT ATG ATG ACA TCA AGA	NM_008084
	Rev-GCC CAG GAT GCC CTT TAG T	

**Table 2:** The incidence of iron supplementation (75 and 150 mg/kg/day *po*) on weight variations, macroscopic lesions and myeloperoxidase activity (MPO) activity in trinitrobenzene sulfonic acid (TNBS)-treated mice. Data are expressed as mean ± SEM.

	Weight Variations After Inflammation (%)	Macroscopic Damage Scores (AU)	MPO Activity (U/mg Protein)
Control	5.03 ± 2.09	0 ± 0	1.24 ± 0.24
Vehicle TNBS	–52.47 ± 20.37 ^**^	5.75 ± 0.75 ^***^	2.85 ± 0.29 ^***^
Iron 75 + TNBS	0.03 ± 0.01	1.41 ± 0.72 ^+++^	1.93 ± 0.15 ^+^
Iron 150 + TNBS	–4.16 ± 0.5	0.20 ± 0.20 ^+++^	1.84 ± 0.17 ^++^

**, *** Significantly different (*p* < 0.01, *p* < 0.001 respectively) from controls. +, ++, +++ Significantly different (*p* < 0.05, *p* < 0.01, *p* < 0.001, respectively) from TNBS-treated mice. TNBS resulted in a significant reduction of weight associated with the marked macroscopic lesions and a clear increase of MPO activity. By contrast, iron supplementation significantly limited the weight loss and the macroscopic lesions, as well as preventing the increase of MPO activity.

## Supplementary Materials

The following are available online at https://www.mdpi.com/2072-6643/11/8/1758/s1, Figure S1: Incidence of lecithin beads microcapsules without ferric pyrophosphate on weight variations (a) and macroscopic lesions (b). Control mice (control), TNBS mice treated with water (TNBS+water) or TNBS mice treated with lecithin beads microcapsules without ferric pyrophosphate (TNBS+vehicle).
