# Supplementary material for: Prevention of Adult Colitis by Oral Ferric Iron in Juvenile Mice Is Associated with the Inhibition of the Tbet Promoter Hypomethylation and Gene Overexpression
Source: Nutrients. 2019 Jul 31;11(8):1758. doi: 10.3390/nu11081758 (PMC6723685; doi:10.3390/nu11081758)
Supplement: Supplementary file 1 [file nutrients-11-01758-s001.pdf]

Figure S1

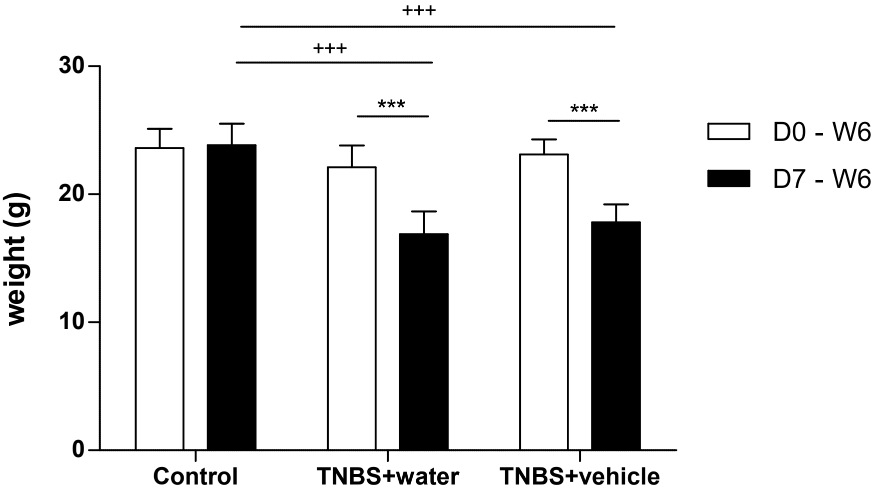

a

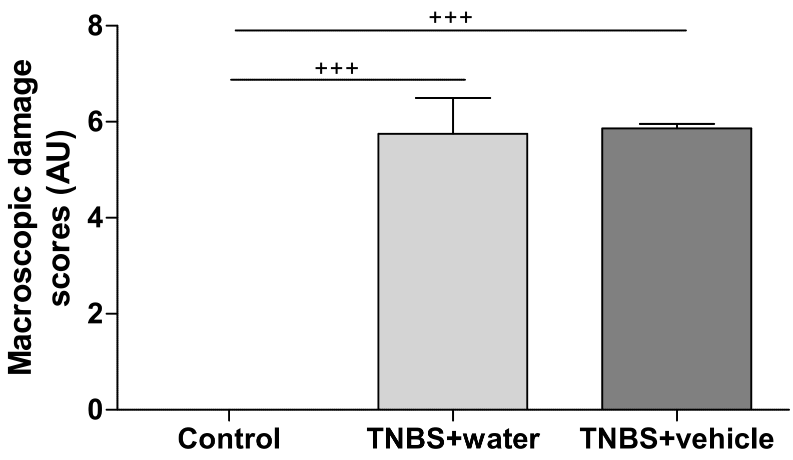

b

Incidence of lecithin beads microcapsules without ferric pyrophosphate on weight variations (**a**) and macroscopic lesions (**b**). Control mice (control), TNBS mice treated with water (TNBS+water) or TNBS mice treated with lecithin beads microcapsules without ferric pyrophosphate (TNBS+vehicle). Data are expressed as mean ± SEM. n=9 for each group.

a *** Significantly different ($P<0.001$) from D0-W6 of same treatment. +++ Significantly different ($P<0.001$) from D7-W6 of Control

b. +++ Significantly different ($P<0.001$) from Control
